# Wearing weighted backpack dilates subjective visual duration: the role of functional linkage between weight experience and visual timing

**DOI:** 10.3389/fpsyg.2015.01373

**Published:** 2015-09-08

**Authors:** Lina Jia, Zhuanghua Shi, Wenfeng Feng

**Affiliations:** ^1^Department of Education, School of Humanities, Jiangnan UniversityWuxi, China; ^2^Department of Psychology, Ludwig-Maximilians-Universität MünchenMunich, Germany; ^3^Department of Psychology, School of Education, SooChow UniversitySuzhou, China

**Keywords:** duration estimation, weight, action affordance, bodily states, embodiment

## Abstract

Bodily state plays a critical role in our perception. In the present study, we asked the question whether and how bodily experience of weights influences time perception. Participants judged durations of a picture (a backpack or a trolley bag) presented on the screen, while wearing different weight backpacks or without backpack. The results showed that the subjective duration of the backpack picture was dilated when participants wore a medium weighted backpack relative to an empty backpack or without backpack, regardless of identity (e.g., color) of the visual backpack. However, the duration dilation was not manifested for the picture of trolley bag. These findings suggest that weight experience modulates visual duration estimation through the linkage between the wore backpack and to-be-estimated visual target. The congruent action affordance between the wore backpack and visual inputs plays a critical role in the functional linkage between inner experience and time perception. We interpreted our findings within the framework of embodied time perception.

## Introduction

Our cognition and perception are grounded in bodily state as well as its interaction with environment (Clark, 1999; Barsalou, 2003). For example, when observers wear a heavy backpack, the geographical slant is likely to be overestimated both in real and virtual hills (Proffitt et al., 1995; Witt and Proffitt, 2007). Similar effects of weight experience have been shown in judgments of spatial distances and monetary values (Witt et al., 2004; Jostmann et al., 2009). When participants threw a heavy ball, the subjective distance was biased by the ball that they threw (Witt et al., 2004). It has been argued (Proffitt, 2006) that such distorted perception reflects the physical energetic costs associated with action plans, as heavy objects, compared to light objects, require more physical strength to act, which is in line with the framework of embodied cognition (Clark, 1999; Barsalou, 2008) that perception, body, and action are tightly linked together.

Not only spatial perception, time perception can also be better understood within the framework of embodiment (Clark, 1999; Droit-Volet et al., 2013; Wittmann, 2013; Maniadakis et al., 2014). Studies have demonstrated that bodily states markedly influence time perception (Yarrow et al., 2001; Droit-Volet and Gil, 2009; Hagura et al., 2012; Shi et al., 2013; Jia et al., 2015). For example, external trains of clicks and intake of drugs (e.g., amphetamine) can change bodily arousal levels, leading to distortions of perceived durations (Maricq et al., 1981; Penton-Voak et al., 1996). Similarly, studies have shown pictures that are more arousing are often perceived longer than low arousal ones (Droit-Volet and Gil, 2009). Voluntary actions or action preparation can also adjust bodily states, affecting subjective time (Effron et al., 2006; Hagura et al., 2012; Maniadakis et al., 2014; Jia et al., 2015). Noted, action-based bodily regulation may overwrite potential influences of affective stimuli on subjective time. For instance, when participants could freely imitate high-arousal facial expressions presented on the screen, the durations of presented angry and happy faces were often overestimated. But such subjective duration expansion diminished when their imitation of the facial expressions was inhibited by holding a pen between their lips (Effron et al., 2006). A recent study (Jia et al., 2015) has also demonstrated that possibility of stimulus–response interaction could change perceived duration of a tactile stimulus.

To explain interactions between subjective time and interoceptive (bodily) states, Craig (2009) proposed awareness theory based on brain imaging studies. According to this theory, the anterior insula cortex unifies meta-representations of homeostatic feeling states that produce a cinemascope ‘image’ of sentient self across time, and subsequently subjective time is estimated through these moments (Craig, 2009; Wittmann et al., 2010; Wittmann, 2013). When a stimulus is related to the survival of body self (e.g., an approaching object toward the observer, see Jia et al., 2015), the inner sentient moments run fast, and subsequently its duration is overestimated. Several recent studies have provided the evidence of this claim (Wittmann et al., 2010; Pollatos et al., 2014; Jia et al., 2015). For instance, the awareness of bodily states influences duration judgments of emotional films (Pollatos et al., 2014). When watching film clips, one group were told to notice their bodily states, whereas the other group were asked to pay attention to the details of film clips to answer several questions later. Afterward, participants recalled the duration of film clips. The results showed that attending to bodily states increased the effects of emotional states on duration judgment compared to attending to clips.

However, the body-related events (e.g., action and emotional) are often accompanied with the changes of arousal, indicated by physiological body response (e.g., increase of skin conductance response and contraction of muscles for threats; Bradley et al., 2001). In other words, bodily states and arousal are hard to separate. In addition, these salient events might capture attention (Vuilleumier, 2005). Another two classic accounts of time perception, the general arousal account and the ‘attention-gate’ theory, can also partially explain time distortions of body-related events using the internal clock model (Gibbon et al., 1984; Gibbon and Church, 1990). According to the internal clock model, the internal clock consists of a pacemaker, a switch, and an accumulator. The switch is located between the pacemaker and the accumulator. When the switch closes, the temporal pulses emitted by the pacemaker are transmitted to the accumulator where the number of pulses decides the length of subjective duration; when the switch opens, the accumulation process stops. Some body-related stimuli would increase the arousal, according to the arousal account, speeding up the pacemaker to emit pulses, and resulting in duration dilation (Hodinott-Hill et al., 2002; Droit-Volet et al., 2004; Nather et al., 2011). By contrast, the ‘attentional-gate’ theory (Block and Zakay, 1997; Zakay and Block, 1997) proposed that attention resources are divided between temporal processing of the clock and non-temporal processing. If a body-related event engages more attention, less attention would be allocated to the timing process in the clock, inducing a loss of some temporal pulses due to the ‘flickering’ open and closed states of the switch. Consequently, duration is underestimated. These three accounts highlight the importance of self-reference, arousal, and attention factors on duration judgment, respectively. The question of which factors play what critical roles in timing has been hot debated recently (Droit-Volet and Meck, 2007; Droit-Volet and Gil, 2009; Maniadakis et al., 2014). Noted, the self-referential process is often coupled with the change of arousal, with the former emphasizing the interaction between the to-be-estimated stimulus and the observer (embodiment). They could commonly contribute to duration distortions in some body-related contexts, although the self-reference and the sensorimotor states seem to play a more important role than affective states (Effron et al., 2006; Nather et al., 2011; Pollatos et al., 2014).

Previous studies concerning embodied timing interpreted that changes of bodily states (e.g., implicit action) caused by target stimuli are critical for duration distortion of the target stimuli (Droit-Volet and Gil, 2009; Wittmann et al., 2010; Shi et al., 2012; Maniadakis et al., 2014). In most cases, the target stimuli and changes of bodily states have some causal relationship, or at least are highly relevant. However, it is unclear whether a functional linkage between the target stimuli and bodily states is necessary for subjective time distortion, or just the change of bodily states already distorts subjective duration. Investigation of such question would provide a new view of interactions between bodily states and timing. One approach is to examine whether and when the duration of a neutral visual stimulus would be distorted in the context of some specific bodily states induced by nonvisual sources, such as weight experience. It has been suggested that weight experience could change bodily states (Proffitt, 2006). For instance, wearing a heavy backpack requires our bodies to afford with more physical efforts relative to wearing a light backpack, and thus different pressure states in the sensory-motor loop might influence temporal judgment by speeding up internal sentient moments (or / and the clock) of weight experience (Craig, 2009; Nather et al., 2011). Given that the neutral visual stimulus is irrelevant to the change of bodily states activated by the weight experience, can the weight experience still impact on visual time judgments in general? If this is the case, it will suggest that the weight experience affects timing by mediating arousal. Alternative, influences of weight experience on visual duration judgments may require some functional linkages, such as by similar action affordance between weight experience and visual target stimulus. According to the theory of affordance (Gibson, 1979), different objects in the environment have different affordances for manipulation. For example, hammer usually affords hitting, knife cutting, and backpack wearing. In line with this view, neurophysiological studies have revealed that even observing the static picture of a manipulable object (e.g., tools) could activate the premotor and parietal motor areas (Chao and Martin, 2000; Grezes and Decety, 2002; Kiefer et al., 2011). The affordance offers the possibility of the linkage between external stimulus and bodily states, which might affect perception and cognition. Studies have shown that recognition of a pictorial object was affected by another pictorial object through the congruent action affordance (Helbig et al., 2006; Kiefer et al., 2011). Based on similar reasoning, we hypothesize that the linkage established by the congruent affordance between weight experience (‘wearing’ behavior) and visual target containing ‘wearing’ affordance (e.g., backpack picture) might be critical for duration distortion of the visual target. Note that arousal and functional linkage are not mutually exclusive, and both can affect time judgment at the same time. Very heavy weight experience may cause great arousing, which may expand subjective duration in general (Gibbon et al., 1984; Droit-Volet and Gil, 2009). Here, we were most interested in whether the functional linkage mediates weight experience and time judgments, thus we only used medium weight in the study.

The present study was designed to investigate whether and how weighted experience, wearing a 5.7 kg backpack, influences visual duration judgments. In particular, whether congruent action affordance between weight experience and visual target plays a key role in subjective time distortion. Participants were asked to judge the duration of computer-presented pictures, either a backpack or trolley bag, while wearing a real weighted or empty backpack. The function of backpack is ‘wearing’, whereas the function of trolley bag is ‘pulling’. Thus, a backpack picture with affordance of ‘wearing’, regardless of its feature (e.g., color, style), might activate its functional linkage to weight experience through the congruent affordance. Then the weighted experience induced by the wore weighted backpack, associated with more energy costs, might dilate subjective duration of the backpack picture via such functional linkage, whereas the ‘pulling’ trolley bag is incongruent in affordance with the ‘wearing’ backpacks, such that the weight experience may have little influence on duration judgments of the visual trolley bag. Alternatively, the general arousal account (Gibbon et al., 1984; Gibbon and Church, 1990) would predict that the duration distortions induced by the wore backpack, if any, would be similar for both backpack and trolley bag pictures. Similarly, the ‘attentional-gate’ theory (Zakay and Block, 1997) would predict underestimated durations, if any, for both the backpack and the trolley bag pictures. This is because if attention is distracted by the wore backpack during the time estimation, less attention for the visual timing task would lead to underestimation. To disassociate these alternative accounts, we conducted three experiments. Experiment 1 compared visual duration estimations of the backpack picture among the conditions of wearing weighted backpack, empty backpack, and no backpack conditions. The backpack depicted in a picture was the same to the wearing one. In Experiment 2, we changed the identity of the visual backpack picture, but remained the same congruent ‘wearing’ affordance. In Experiment 3, we changed the backpack picture to a trolley bag picture, which has different action affordance meanings (‘pulling’) from the wore backpack (‘wearing’).

## Materials and Methods

### Participants

Fifty-five students from Jiangnan University took part in the experiments (18, 19, and 18 in Experiments 1, 2, and 3, respectively; 37 female; mean age = 20.7, *SD =* 2.7). The numbers of females were 11, 12, and 14 in Experiments 1, 2, and 3, respectively. All participants had normal or corrected-to-normal visual acuity and no somatosensory disorders. All participants were naive to the purpose of experiments. The experiments were approved by the ethics committee of Jiangnan University. Informed consent in accordance with the Declaration of Helsinki was obtained from each participant before the start of the experiment.

### Stimuli and Apparatus

**FIGURE 1:**
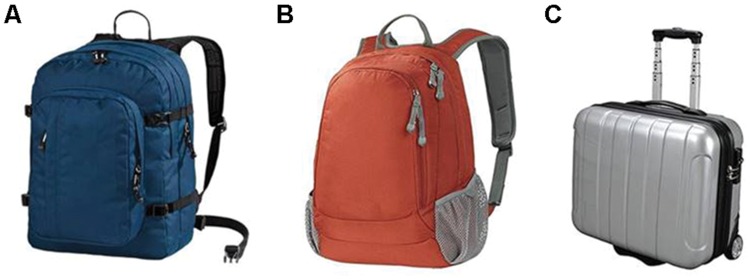
**Visual pictures used in three experiments for duration judgments. (A)** The blue backpack picture presented in Experiment 1, and participants wore this type of backpack in all experiments; **(B)** The orange backpack picture used in Experiment 2; **(C)** The small trolley bag picture used in Experiment 3.

The experiments were conducted in an isolated cabin with dim lit environment. Visual stimuli were presented on a 21-inch CRT monitor with a refresh rate of 100 Hz. Visual stimuli consisted of the following pictures: blue and orange backpacks (12 cm × 9 cm), small gray business trolley bag (10 cm × 10 cm, see **Figure 1**). Participants were asked to keep standing and holding a light response box during blocks. The viewing distance was kept at 57 cm. Visual stimuli presentation was controlled by Matlab program using Psychophysics Toolbox (Brainard, 1997).

In each trial, the to-be-estimated visual duration was the exposure time of a picture, which could be a blue backpack (Experiment 1), an orange backpack (Experiment 2), or a gray trolley bag (Experiment 3). During all experiments, participants wore a blue backpack (44 cm × 32 cm × 35cm) depicted in **Figure 1A**. Prior to the experiment, participants were told that the weights of the blue backpack (**Figure 1A**), orange backpack (**Figure 1B**), or small trolley bag (**Figure 1C**) in pictures were the same as the wore blue backpack (weighted or empty).

### Experimental Procedure

A classic temporal bisection task was used in the experiments. Participants were first trained to discriminate two visual anchor durations: a short one (200 ms) and a long one (600 ms). The anchor stimulus was a white rectangle (12 cm × 9 cm for Experiments 1 and 2; 10 cm × 10 cm for Experiment 3), same size as the pictures used in the experiments. The training session ended when participants reached 100% accuracy of discrimination for consecutive 20 trials.

**FIGURE 2:**
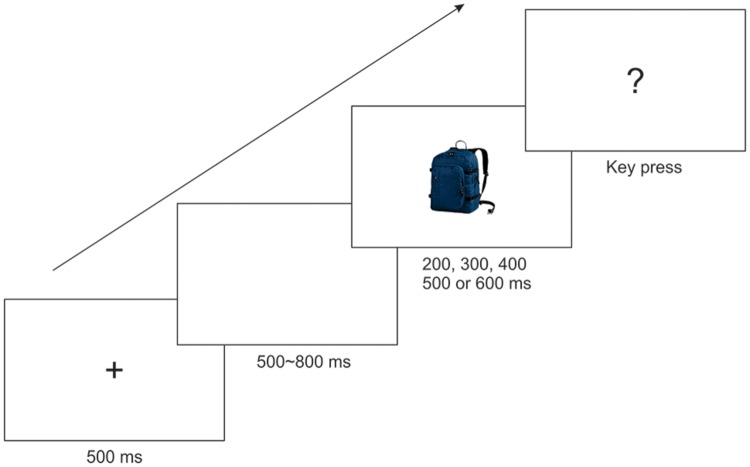
**Illustration of a trial sequence.** Note that the target picture was a blue backpack, an orange backpack, and a trolley bag in Experiments 1, 2, and 3, respectively.

In the subsequent test session, illustrated in **Figure 2**, each trial started with a fixation cross for 500 ms, followed by a blank display randomly for 500∼800 ms. Then a target picture (backpack in Experiments 1 and 2, trolley bag in Experiment 3) was presented for a given probe duration, randomly selected from 200, 300, 400, 500, or 600 ms. After the picture presentation, a question mark was shown to prompt for a response. Participants had to judge whether the duration of the picture was closer to the short anchor (200 ms) or the long anchor (600 ms) as accurately as possible by pressing the left or right key on the response box, respectively. The inter-trial interval (ITI) varied randomly from 1000 to 1500 ms.

The test session consisted of three conditions of wearing weights block-wisely: the weighted backpack (5.7 kg), empty backpack (0.7 kg), and no backpack (baseline) conditions. Each weight condition was repeated twice, and randomly intermixed with the other conditions. Within each block, five probe durations were repeated randomly for 10 times, yielding 50 trials per block. Thus, the test session consisted of 300 trials. To refresh participants about the short and long anchors, each of the two anchors was presented for five times at the beginning of each block. Participants took a rest about 2 min by taking off the backpack between blocks. The length of the test session was around 50 min.

After the test session, participants were asked to rate the valence and arousal using the paper sheet of the affective-rating Self-Assessment-Manikin (SAM) in order to compare the arousal levels among three conditions of wearing weights. The SAM evaluation is 9-point scales rating, ranging from sad to pleasant for the ‘valence’ and from calm to activated for the ‘arousal’ (Bradley and Lang, 1994). To make sure that participants understood the meanings of 9 points on valence and arousal scales, respectively, they were presented with the detailed instruction before their evaluation.

## Results

The proportions of ‘long’ responses for the five probe durations were calculated and fitted by a logistic function for each participant at each weight condition. The points of subjective equality (PSEs) of the temporal bisection were then estimated corresponding to the duration at the 50th percentile of the fitted curves. To measure the sensitivity of duration judgments, the just-noticeable differences (JNDs) were estimated by taking half the difference in durations between the 25th and 75th percentiles (see detailed method in Shi et al., 2012). Repeated-measures ANOVAs with wearing weight as factor were conducted separately on the PSEs and JNDs in all experiments, and then further LSD contrast tests were performed to see the significant differences among conditions of wearing weights. Similar ANOVAs were applied for subjective arousal ratings.

### Duration Judgment

**FIGURE 3:**
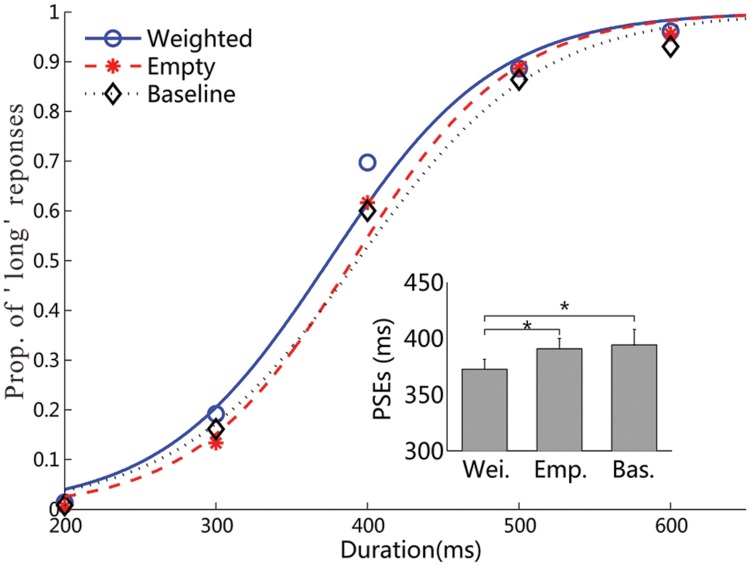
**Results of Experiment 1.** Mean proportions of ‘long’ responses in the visual duration bisection task, and the fitted psychometric functions, are plotted against the probe durations for the three weight conditions. The inset figure shows the mean PSEs, and related standard errors, for the three conditions (all ^∗^*p* < 0.05).

Experiment 1 examined the influences of wearing a backpack on the duration judgment of the same backpack picture. **Figure 3** shows the psychometric curves of the visual-duration bisection task for the weighted backpack, empty backpack, and baseline conditions, respectively. The mean PSEs (±SE) were 373 ± 9, 391 ± 9, and 394 ± 13 ms for the weighted backpack, empty backpack, and baseline conditions (**Table 1**). Repeated measures ANOVA revealed a significant influence of wearing weights on the visual duration judgment, *F*(2,34) = 3.66, *p* < 0.05, $\eta_{p}^{2}$ = 0.18. The further post-hoc contrast tests showed significant differences in PSEs between the weighted and empty backpack conditions (difference: 18 ms, *p* < 0.05), and between the weighted backpack and baseline conditions (difference: 21 ms, *p* < 0.05), but not between the empty backpack and baseline conditions (*p* = 0.70). The JNDs (±SE) were 53 ± 6, 50 ± 4, and 55 ± 3 ms for the weighted backpack, empty backpack, and baseline conditions (**Table 1**). A repeated-measures ANOVA failed to show any significant difference on JNDs among these three conditions, *F*(2,34) = 0.69, *p* = 0.51, $\eta_{p}^{2}$ = 0.04.

**Table 1:** Mean of points of subjective equality (PSEs) and just-noticeable differences (JNDs) for three weight conditions across all experiments (ms).

	PSE(±SE)			JND(±SE)		
	Weighted	Empty	Baseline	Weighted	Empty	Baseline
Experiment 1	373(9)	391(9)	394(13)	53(6)	50(4)	55(3)
Experiment 2	388(11)	412(10)	404(13)	53(4)	62(5)	54(3)
Experiment 3	398(12)	395(13)	401(12)	55(3)	58(4)	51(4)

**FIGURE 4:**
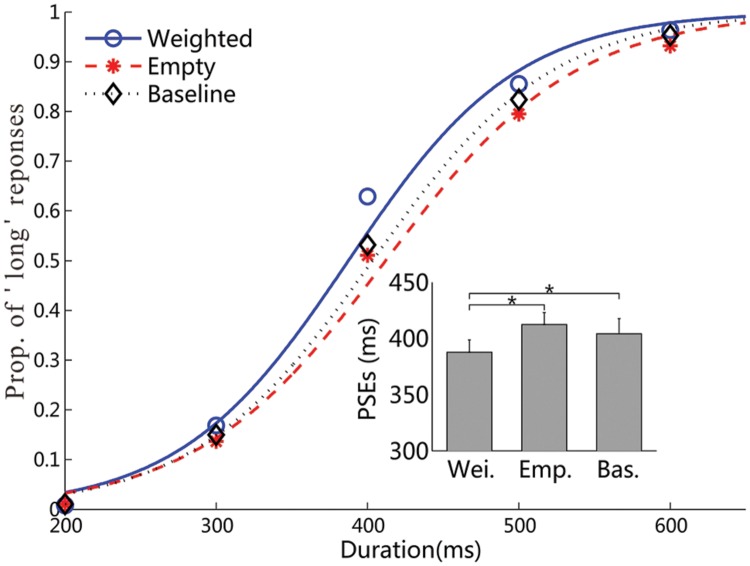
**Results of Experiment 2.** The psychometric functions are fitted for the three weight conditions. The inset figure shows the mean PSEs (SE) for the three conditions (all ^∗^*p* < 0.05).

Experiment 2 changed the identity of the backpack picture, yielding similar results as those of Experiment 1 (**Figure 4**). The mean PSEs (±SE) were 388 ± 11, 412 ± 10, and 404 ± 13 ms for the weighted, empty backpacks and baseline conditions, respectively (**Table 1**). The ANOVA revealed that the influence of the feeling of weight on visual duration judgments was significant, *F*(2,36) = 3.5, *p* < 0.05, $\eta_{p}^{2}$ = 0.16. The post-hoc contrasts showed significant differences in PSEs between the weighted and empty backpack conditions, the weighted and baseline conditions, respectively (differences: 24 and 16 ms, both *p* < 0.05), but no significant difference between the empty backpack and baseline conditions (*p* = 0.44). A further ANOVA on discrimination sensitivity (JNDs) showed a marginal significance among three conditions, *F*(2,36) = 3.3, *p* = 0.05, $\eta_{p}^{2}$ = 0.16. Further contrast tests indicated that the JND in the weighted backpack condition was significantly lower than that in the empty backpack condition (*p* < 0.05), while no differences were shown in other comparison conditions (weighted backpack vs. baseline: *p* = 0.83; empty backpack vs. baseline: *p* = 0.07).

**FIGURE 5:**
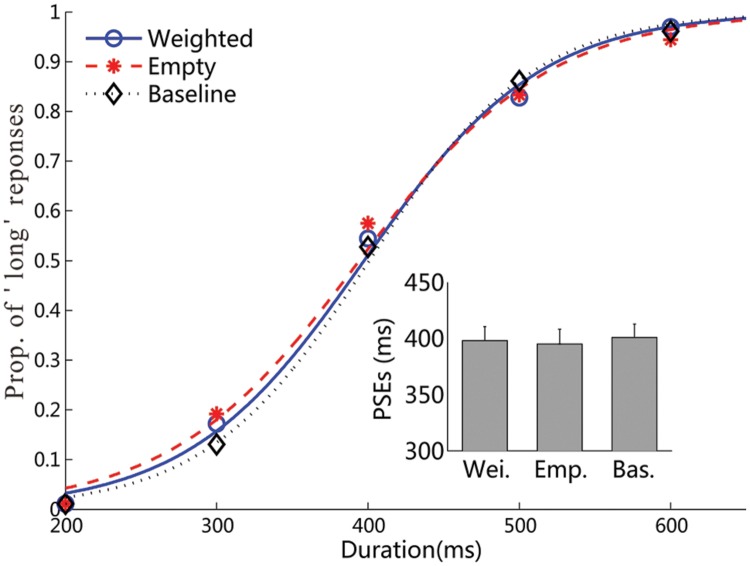
**Results of Experiment 3.** The psychometric functions are fitted for the three weight conditions. The inset figure shows the mean PSEs (SE) for the three conditions.

Experiment 3, on the other hand, revealed different outcomes (**Figure 5**). The mean PSEs (±SE) were in similar magnitudes for the three conditions: 398 ± 12, 395 ± 13, and 401 ± 12 ms for the weighted, empty backpacks and baseline, respectively (**Table 1**), and failed to reveal any main effect of perceiving weight on the visual duration judgment, *F*(2,34) = 0.28, *p* = 0.76, $\eta_{p}^{2}$ = 0.02. Similar to the previous two experiments, the JNDs (±SE) also failed to show any significant difference among three conditions, *F*(2,34) = 1.23, *p* = 0.31, $\eta_{p}^{2}$ = 0.07.

### Assessment of Arousal

Given that subjective ratings of arousal were similar across three experiments, we collapsed arousal ratings across three experiments for the weighted backpack, empty backpack, and baseline conditions. The total results showed that the subjective ratings of arousal significantly differed among the three conditions (Greenhouse–Geisser Correction: *F*(2,108) = 5.14, *p* < 0.05, $\eta_{p}^{2}$ = 0.09). The further contrast tests showed that both weighted (mean 4.95) and empty (mean 4.72) backpacks were rated to be more arousing than the baseline (mean 4.53) (both *p* < 0.05), but there was no evidence of significant difference between the weighted and empty backpack conditions (*p* = 0.12).

## Discussion

The present study examined how wearing a medium weighted backpack modulated visual duration judgments. We found that wearing a weighted backpack (5.7 kg) lengthened subjective duration of a backpack picture, regardless of the identity of the backpack. In contrast, weight experience failed to impact duration judgments of a trolley bag picture. The findings suggest that the effect of weight experience on visual duration judgments depends on a functional link between weight experience and the to-be-estimated picture.

Our findings of differential impacts of weight experiences on visual duration judgments could hardly be explained by the arousal-based account (Gibbon et al., 1984) or the attentional-gating theory (Zakay and Block, 1997). SAM evaluation showed that the rated arousal levels for the weighted and empty backpacks were higher than baseline condition. According to the arousal account, visual duration should be expanded, if any, across all experiments for wearing backpack compared to not wearing backpack. However, the duration expansion effect was only revealed for the weighted backpack condition in Experiments 1 and 2 where the visual targets were backpack pictures, but not in Experiment 3 where the visual target was a trolley bag. Alternatively, attentional-gating theory would predict that attention shifts away from the duration judgment task, if any, to the weight experience, the visual duration would be underestimated, not overestimated. However, such underestimation was not observed in our experiments. Moreover, both arousal and attention accounts would predict reduced temporal sensitivity of temporal bisection in the wore backpack condition compared to the baseline condition, which was not the case in our study as we failed to find their significant differences in JNDs. It should be pointed out, we do not argue that attention and arousal states cannot affect duration judgments (in fact, they do significantly influence duration judgments shown in other studies), rather we suggest merely using attention and arousal states cannot explain the present findings.

Alternatively, our findings can be better explained by the awareness theory based on the embodiment framework (Craig, 2009; Wittmann, 2013; Maniadakis et al., 2014), according to which time perception is an accumulation process of self-related moments (Craig, 2009; Wittmann et al., 2010). In line with this view, recent studies have shown the modulation of near-body arousing stimuli on duration judgment (Effron et al., 2006; Wittmann and van Wassenhove, 2009; Shi et al., 2012; Jia et al., 2015). The bodily experience initiated by the to-be-estimated stimuli with near-body meaning might speed up inner sentient ‘moments’, leading to duration dilation (Wittmann et al., 2010; Pollatos et al., 2014). It should be noted that in most previous studies bodily states are directly manipulated by affective stimuli or related actions, which are closely related to duration judgments. The present study, on the other hand, provides the first evidence that the functional linkage between timing task and self-referential process is important for the interactions between visual duration judgments and weight experience. The activity of weight pressure was irrelevant to the to-be-estimated target (here the backpack or trolley bag picture), but they could be automatically linked through congruent action affordance. Specifically, when the visual target was the ‘wearable’ backpack but not a trolley bag, similar to what they wore in affordance, the inner sentient moments for the weight experience and visual estimation were possibly merged together, biasing the time estimation of the visual input in the weighted backpack condition. By contrast, when the visual input had different action affordances (e.g., ‘pulling’ of the trolley bag), the inner sentient moments for the weight experience and visual stimulation were likely to be separated, resulting in no effect of weight experience on visual duration judgments. Similar congruency effect of affordance has been demonstrated in response performance (Chen and Bargh, 1999; Alexopoulos and Ric, 2007). The present study extended the affordance congruency effect to duration judgments.

One might argue, however, the same category (‘backpack’), rather than congruent affordance, between weight experience and visual blue or orange backpacks, contributed to the linkage. Both the visual blue and orange backpacks can be categorized as ‘backpack’, but trolley bag cannot. Thus, the category linkage between visual backpacks and the wore backpack might be proposed to induce the impact of weight on visual timing. Gibson (1979) assumed that the same category (defined by the common features) just means a conceptual ‘family resemblance’ and does not correspond to the congruent affordance. We believe affordance congruency, rather than same category, provides direct linkage between bodily states and time process. First, it has been shown that similar action affordance, not the same category, modulated task performance (Helbig et al., 2006; Weatherford et al., 2015). For example, recognition of the target object following a prime object was facilitated when two objects had the congruent action affordance, although could be classified differently (e.g., pan–dustpan) (Helbig et al., 2006). Second, objects with congruent affordance elicited common neural activities related to motor (Kiefer et al., 2011; Sim et al., 2015), which provides potential mechanism underlying the interaction between perception and bodily states. On this ground, we believe that the congruent affordance between the visual input and weight experience contributed to duration distortion. Still, physiological measures should be used in future work to identify a neural linkage between weight experience and visual timing through the congruent affordance.

## Conclusion

The present research extends the evidence of embodied timing by revealing that wearing a weighted backpack dilates subjective visual duration through a functional linkage. The congruent action affordance between wearing behavior of weight and visual target is critical for such functional role of weight experience on visual timing. Note that we only applied three types of stimuli in visual modality. Thus, future work should expand stimuli to more general categories, and focus on influences of various types of action linkage between weight experience and duration estimation by using different types of sensory inputs, not limited to visual modality.

## Conflict of Interest Statement

The authors declare that the research was conducted in the absence of any commercial or financial relationships that could be construed as a potential conflict of interest.
